# Surgical treatment of mechanical bowel obstruction: characteristics and outcomes of geriatric patients compared to a younger cohort

**DOI:** 10.1007/s00384-022-04152-4

**Published:** 2022-05-05

**Authors:** Christian J. J. Paul, Jonas Dohmen, Cornelius J. van Beekum, Maria A. Willis, Lara Braun, Jörg C. Kalff, Arnulf G. Willms, Tim O. Vilz

**Affiliations:** 1 Department of General, Visceral, Thoracic and Vascular Surgery, University Hospital Bonn, Bonn, Germany; 2 Department of General, Visceral and Thoracic Surgery, German Armed Forces Central Hospital Koblenz, Koblenz, Germany

**Keywords:** Mechanical bowel obstruction, Geriatric patients, Large bowel obstruction, Small bowel obstruction, Malignant bowel obstruction, Emergency surgery

## Abstract

**Purpose:**

Mechanical bowel obstruction (MBO) is one of the most common indications for emergency surgery. Recent research justifies the method of attempting 3–5 days of nonoperative treatment before surgery. However, little is known about specific characteristics of geriatric patients undergoing surgery compared to a younger cohort. We aimed to analyze patients with MBO that required surgery, depending on their age, to identify potential targets for use in the reduction in complications and mortality in the elderly.

**Methods:**

Thirty-day and in-hospital mortality were determined as primary outcome. We retrospectively identified all patients who underwent surgery for MBO at the University Hospital of Bonn between 2009 and 2019 and divided them into non-geriatric (40–74 years, *n* = 224) and geriatric (≥ 75 years, *n* = 88) patients, using the chi-squared-test and Mann–Whitney *U* test for statistical analysis.

**Results:**

We found that geriatric patients had higher 30-day and in-hospital mortality rates than non-geriatric patients. As secondary outcome, we found that they experienced a longer length of stay (LOS) and higher complication rates than non-geriatric patients. Geriatric patients who suffered from large bowel obstruction (LBO) had a higher rate of bowel resection, stoma creation, and a higher 30-day mortality rate. The time from admission to surgery was not shown to be crucial for the outcome of (geriatric) patients.

**Conclusion:**

Geriatric patients suffering from mechanical bowel obstruction that had to undergo surgery had higher mortality and morbidity than non-geriatric patients. Especially in regard to geriatric patients, clinicians should treat patients in a risk-adapted rather than time-adapted manner, and conditions should be optimized before surgery.

**Supplementary information:**

The online version contains supplementary material available at 10.1007/s00384-022-04152-4.

## Introduction

Mechanical bowel obstruction (MBO) is one of the most common indications for emergency surgery. In North America, more than 300,000 admissions to hospital are due to small bowel obstruction (SBO) each year [1, 2], which annually cause over USD $2 billion worth of inpatient costs [3].

During the last couple of years, a lot of research has been carried out in regard to MBO. Although latest research has shown that a trial of at least 3–5 days of nonoperative treatment is justified in the absence of urgent indications for surgery [4–9], MBO plays a major role in emergency medicine and accounts for 20–50% of emergency surgeries performed [10–12]. MBO is a severe illness that is associated with a mortality of 10–30% [1, 13–15]. Some reports highlight an increase in mortality after 3 days or more of nonoperative treatment before surgery [2, 13, 16–18], and some reports recommend that MBO should be treated surgically within 24 h [19–22]. Thus, patients suffering from MBO who are treated surgically are a group of special interest.

Major risk factors for the development of MBO are prior abdominal surgery, especially lower abdominal or pelvic surgery, malignant diseases, and recurrent diverticulitis, and these risk factors should be identified when the medical history is taken [6, 8]. In identifying patients who require surgery, abdominal computed tomography (CT) with oral and intravenous contrast medium is the gold standard in the diagnosis of MBO [8, 23] and should be performed quickly [24–26]. Some recent studies point out that the sensitivity of ultrasound in the diagnosis of SBO is approximately 92% [27, 28], which suggests that ultrasound could be an easily accessible tool for initial imaging in patients suffering from abdominal pain.

However, there are only few data that examine the impact of age on the time from presentation to the emergency department (ED) to surgery, on the surgical procedure and on the morbidity and mortality rates thereafter. Krause et al. [29] showed that the time from admission to surgery did not differ between the observed age cohorts, whereas van Beekum et al. [30] demonstrated a reduced time period in younger patients, but in both trials, there was no further analysis of the geriatric patients in terms of special characteristics influencing the outcome of this group. As the population is continuously aging [31, 32], further research, especially on the particular characteristics of the elderly, is essential.

In our trial, we aimed to analyze patients who suffered from MBO and had an indication for surgical treatment depending on their age. We aimed to examine whether there were differences between geriatric and non-geriatric patients in terms of the etiology of MBO and the time from presentation to the ED to surgery, and whether such differences influence the 30-day and the in-hospital mortality as primary outcome and the LOS at ICU and in hospital, the complications classified by the classification system of Clavien-Dindo (C-D) [33], and the need for redo surgery, bowel resection, or stoma creation as secondary outcome. In addition, we aimed to examine notable characteristics in the geriatric group that influenced their outcomes in order to identify potential targets for use in the reduction in complications and mortality in the elderly in the context of an aging population.

## Methods

We retrospectively identified all patients that presented to the ED and underwent abdominal surgery due to MBO at the University Hospital of Bonn between 01/01/2009 and 10/31/2019. Patients suffering from MBO with successful conservative therapy were not included. We divided the patients into two groups based on their age at the time of surgery, into non-geriatric patients (40–74 years), and geriatric patients (≥ 75 years). We chose a cut-off age of 75 years to define elderly patients because it has been reported as the at-risk elderly population for adverse outcome in the large American College of Surgeons-National Surgical Quality Improvement Program (NSQIP) cohort of patients undergoing major GI surgery [34]. We received the data from patients’ medical records, physicians’ letters, surgical reports, and anesthesiologic protocols.

We analyzed the time between presentation to the ED and surgery, pre-existing diseases, the etiology, and the localization of bowel obstruction, the need for bowel resection, and stoma creation. The postoperative course was evaluated using the classification system of C-D, the need for redo surgery and the mortality rates, divided into 30-day and in-hospital mortality rates. Furthermore, we analyzed the LOS and the LOS in the intensive care unit (ICU) of non-geriatric vs. geriatric patients. Moreover, the group of geriatric patients was further analyzed regarding the effect of predictive factors on the outcome of these patients.

We considered 30-day mortality and in-hospital mortality as primary outcome and LOS, complications classified by C-D, and the need for redo surgery, bowel resection, or stoma creation as secondary outcome.

For statistical analysis, we used SPSS version 26 (IBM SPSS, Chicago, IL, USA). Results are presented as median (~ x). The chi-squared-test and Mann–Whitney *U* test were used to compare the frequency distribution between the patient groups, and to further analyze the geriatric group, *p* values less than 0.05 were considered statistically significant.

## Results

### Demographics (patient collective)

In total, we identified 349 patients who underwent surgery for MBO. A total of 37 patients were younger than 40 years old. In total, 224 patients were between 40 and 74, and 88 patients were at least 75 years old; these two groups were included in further analyses (*n* = 312).

### Outcome

#### Primary outcome

The 30-day mortality rate, as well as the in-hospital mortality rate, was higher in the geriatric patients, implying that they had a worse primary outcome than non-geriatric patients (Table 1).

**Table 1:** Postoperative course

	**40–74 years**	** ≥ 75 years**	***p*-value**
	*n* = *204*	*n* = *72*	
**LOS at ICU (h)**	~ x = 17	~ x = 49	0.002
**LOS (h)**	~ x = 364	~ x = 498	0.003
	*n* = *224*	*n* = *88*	
**Redo surgery**	70 (31.3%)	31 (35.2%)	ns
**Postoperative complications (C-D)**			
0	37 (16.5%)	1 (1.1%)	< 0.001
I	20 (8.9%)	7 (8.0%)	ns
II	51 (22.8%)	26 (29.5%)	ns
IIIA	37 (16.5%)	14 (15.9%)	ns
IIIB	51 (22.8%)	9 (10.2%)	0.011
IVA	7 (3.1%)	11 (12.5%)	0.001
IVB	1 (0.4%)	4 (4.5%)	0.009
V	20 (8.9%)	16 (18.2%)	0.021
Need for bowel resection	107 (47.8%)	44 (50%)	ns
Need for stoma creation	71 (31.7%)	24 (27,3%)	ns
**30-day mortality**	11 (4.9%)	14 (15.9%)	0.001
**In-hospital mortality**	20 (8.9%)	16 (18.2%)	0.021

#### Secondary outcome

The overall rate of postoperative complications, classified by the C-D classification system, was higher in the group of geriatric patients. Compared to the group of non-geriatric patients (Table 1). The number of redo surgeries carried out did not differ significantly between the two groups. Furthermore, bowel resection was necessary in half of the cases, while a stoma was created in about one-third of the cases in both groups. Additionally, the LOS in hospital, as well as the LOS at the ICU, were longer in the geriatric group than in the non-geriatric group.

### Admitting characteristics for patients with bowel obstruction

Our analyses demonstrated that especially in regarding to the rates of cardiac, vascular, metabolic, and neurologic diseases, these rates were significantly higher in the group of geriatric patients. In the group of non-geriatric patients, we showed that the rate of pre-existing malignant diseases was significantly higher than in the group of geriatric patients (Table 2).

**Table 2:** Admitting characteristics for patients with bowel obstruction

	**40–74 years**	** ≥ 75 years**	***p*-value**
	*n* = 224	*n* = 88	
**Male**	115 (51.3%)	44 (50.0%)	ns
**Pre-existing disease**			
Cardiac	68 (30.4%)	51 (58.0%)	< 0.001
Vascular	129 (57.6%)	77 (87.5%)	< 0.001
Pulmonary	27 (12.1%)	18 (20.5%)	ns
Renal	22 (9.8%)	13 (14.8%)	ns
Hepatic	12 (5.4%)	1 (1.1%)	ns
Neurologic	40 (17.9%)	26 (29.5%)	0.023
Metabolism (diabetes, obesity…)	62 (27.7%)	39 (44.3%)	0.005
Malignant	88 (39.3%)	20 (22.7%)	0.006
Inflammatory bowel disease	12 (5.4%)	1 (1.1%)	ns
Miscellaneous	6 (2.7%)	0 (0%)	ns
**ASA score**			
I	0 (0%)	0 (0%)	
II	78 (34.8%)	8 (9.1%)	< 0.001
III	120 (53.6%)	77 (87.5%)	< 0.001
IV	26 (11.6%)	3 (3.4%)	0.025
V	0 (0%)	0 (0%)	ns
**Time between presentation to the ED and surgery (h)**	~ x = 23.4	~ x = 14.6	ns
	*n* = 186	*n* = 71	
**Time from presentation to the ED and surgery (h)—SBO**	~ x = 22.6	~ x = 11.7	0.015
	*n* = 38	*n* = 17	
**Time from presentation to the ED and surgery (h)—LBO**	~ x = 26.3	~ x = 21.0	ns

Furthermore, we significantly demonstrated that the non-geriatric patients were more commonly classified with an American Society of Anesthesiologists (ASA) score of II or IV than the geriatric patients, whereas the geriatric patients were more commonly classified with an ASA score of III than the non-geriatric patients, meaning that geriatric patients were in general at higher preoperative risk, with exception of the few critically ill patients classified with an ASA score of IV in the non-geriatric patients.

### Time from presentation to the ED to surgery

As our results show, the time from presentation to the ED to surgery was—regarding patients who suffered from SBO—significantly shorter in the geriatric group than in the non-geriatric group.

In general, and in patients who suffered from large bowel obstruction (LBO), there was no significant difference concerning the time from presentation to the ED to surgery between geriatric and non-geriatric patients.

### Etiology and intraoperative findings

In both groups, adhesions were the main cause for MBO. Malignancies also played a crucial role in causing MBO, while “other malignancies” (peritoneal carcinosis, gastrointestinal stromal tumor, ovarian/endometrial cancer and prostate cancer) showed a higher impact than colorectal carcinoma (CRC) (Table 3). The two groups differed significantly in the frequency of adhesions, gallstone ileus, and inflammation.

**Table 3:** Intraoperative findings

	**40–74 years**	** ≥ 75 years**	***p*-value**
	*n* = *224*	*n* = *88*	
**Localization of bowel obstruction**			
Small bowel	186 (83.0%)	71 (80.7%)	ns
Large bowel	38 (17.0%)	17 (19.3%)	ns
**Cause of bowel obstruction**			
Adhesions	108 (48.2%)	54 (61.4%)	0.036
All malignancies	65 (29.0%)	18 (20.5%)	ns
CRC	18 (8.0%)	5 (5.7%)	ns
Other malignancies	47 (21.0%)	13 (14.8%)	ns
Hernia	15 (6.7%)	4 (4.5%)	ns
Volvulus	6 (2.7%)	3 (3.4%)	ns
Intussusception	3 (1.3%)	2 (2.3%)	ns
Gallstone ileus	0 (0%)	3 (3.4%)	0.005
Inflammation	10 (4.5%)	0 (0%)	0.044
Exposure to radiotherapy	4 (1.8%)	0 (0%)	ns
Miscellaneous	13 (5.8%)	4 (4.5%)	ns

In both groups, a major part of MBO was localized in the small bowel.

In addition, we examined whether there were differences between the geriatric and non-geriatric patients, separated by SBO and LBO (see additional file 1, supplementary Tables 1 and 2). We found that the intraoperative findings regarding patients who suffered from SBO or LBO did not differ significantly between geriatric and non-geriatric patients; the only difference was that geriatric patients who suffered from SBO had a higher rate of gallstone ileus (see additional file 1, supplementary Table 1).

### Further analysis of the geriatric patients

Additionally, we took a closer look at the group of geriatric patients to identify remarkable characteristics within this group (*n* = 88). When we analyzed the LOS in this group, we excluded the 16 patients who died in the hospital (*n* = 72), as shown in Table 4.

**Table 4:** Further analysis of the geriatric patients

Variable	Characteristic	LOS ICU (h)	LOS (h)	Need for bowel resection	Need for stoma creation	Redo surgery	30-day mortality	In hospital mortality
		*n* = 72	*n* = 72	*n* = 88	*n* = 88	*n* = 88	*n* = 88	*n* = 88
Sex	Male	~ x = 69	~ x = 617	24 (54.5%)	16 (36.4%)	18 (40.9%)	8 (18.2%)	9 (20.5%)
	Female	~ x = 15	~ x = 466	20 (45.5%)	8 (18.2%)	13 (29.5%)	6 (13.6%)	7 (15.9%)
	*p*-value	0.021	ns	ns	ns	ns	ns	ns
Pre-existing cardiac disease	No cardiac disease	~ x = 15	~ x = 408	20 (54.1%)	10 (27.0%)	10 (27.0%)	4 (10.8%)	4 (10.8%)
	Cardiac disease	~ x = 53	~ x = 622	24 (47.1%)	14 (27.5%)	21 (41.2%)	10 (19.6%)	12 (23.5%)
	*p*-value	0.016	0.049	ns	ns	ns	ns	ns
Pre-existing malignant disease	No malignant disease	~ x = 52	~ x = 441	32 (47.1%)	12 (17.6%)	19 (27.9%)	11 (16.2%)	13 (19.1%)
	Malignant disease	~ x = 14	~ x = 672	12 (60.0%)	12 (60.0%)	12 (60.0%)	3 (15.0%)	3 (15.0%)
	*p*-value	ns	0.021	ns	< 0.001	0.008	ns	ns
Cause of bowel obstruction	Adhesions	~ x = 51	~ x = 492	25 (46.3%)	7 (13.0%)	18 (33.3%)	7 (13.0%)	9 (16.7%)
	No adhesions	~ x = 21	~ x = 527	19 (55.9%)	17 (50.0%)	13 (38.2%)	7 (20.6%)	7 (20.6%)
	*p*-value	ns	ns	ns	< 0.001	ns	ns	ns
Cause of bowel obstruction	All malignancies	~ x = 7	~ x = 656	10 (55.6%)	12 (66.7%)	9 (50.0%)	4 (22.2%)	4 (22.2%)
	No malignancies	~ x = 53	~ x = 462	34 (48.6%)	12 (17.1%)	22 (31.4%)	10 (14.3%)	12 (17.1%)
	*p*-value	0.029	ns	ns	< 0.001	ns	ns	ns
Localization of bowel obstruction	Small bowel	~ x = 45	~ x = 492	31 (43.7%)	14 (19.7%)	22 (31.0%)	8 (11.3%)	10 (14.1%)
	Large bowel	~ x = 69	~ x = 668	13 (76.5%)	10 (58.8%)	9 (52.9%)	6 (35.3%)	6 (35.3%)
	*p*-value	ns	ns	0.015	0.001	ns	0.025	ns

We found that the male geriatric patients stayed significantly longer in the ICU than the female geriatric patients. Furthermore, the geriatric patients that suffered from a cardiac disease stayed significantly longer in the ICU, as well as in hospital, than the geriatric patients without cardiac diseases. Patients who suffered from a pre-existing malignant disease had significantly higher rates of stoma creation and redo surgery and stayed longer in hospital than geriatric patients who did not suffer from a pre-existing malignant disease (Table 4).

If the bowel obstruction was caused by adhesions, the geriatric patients showed significantly lower rates of stoma creation than the geriatric patients who suffered from bowel obstruction due to another cause. Geriatric patients who suffered from malignant bowel obstruction (MABO) stayed for a shorter time in the ICU, but had a higher need for redo surgery than patients who suffered from MABO due to another cause.

The geriatric patients who suffered from LBO showed significantly higher rates of bowel resection and stoma creation, as well as a higher 30-day mortality rate, than the patients who suffered from SBO.

## Discussion

In our study, a retrospective analysis of two patient cohorts, divided into geriatric and non-geriatric patients, was performed.

As primary outcomes, we observed the 30-day and in-hospital mortality rates in our patients which were significantly higher in the geriatric than in the non-geriatric patients. This is similar to Krause et al. [29] and van Beekum et al. [30], who observed in-hospital-mortality rates of 0.0% and 3.2%, respectively, in the younger, and 9.1%, and 23.5%, respectively, in the older cohort; Krause et al. only included patients suffering from SBO, whereas van Beekum et al. also included patients suffering from LBO. Furthermore, the cohorts were grouped differently in the two studies: Krause et al. [29] included patients aged 65 years or older in the geriatric group, whereas van Beekum et al. [30] included patients aged 80 years or older in the geriatric group.

Compared to these two trials, and compared to the recent literature, our in-hospital-mortality rate of 8.9% in the non-geriatric and 18.2% in the geriatric patients is average. In SBO patients, Long et al. [3] described an overall mortality rate of 7–14% in the elderly, contrary to < 3% in younger patients. The average 30-day mortality rate for SBO is between 5 and 30% [1, 13–15, 35], whereas the average mortality rate for LBO is between 10 and 20% [14]. If the bowel obstruction is caused by a malignancy, e.g., peritoneal carcinosis, the average mortality rate is between 6 and 32% [36–39]. Some trials show that the risk of emergency gastrointestinal surgery is higher in the elderly [40, 41]; some studies also suggest that age itself is a risk factor for higher mortality rates in emergency gastrointestinal surgery [4, 14, 42, 43].

As secondary outcomes, we regarded the rate of bowel resection and stoma creation; there was no difference between the geriatric and the non-geriatric group. Furthermore, the rate of bowel resection in both groups was rather high compared to the literature, which reports a rate of 30–45% in regard to bowel resection [8, 15, 29, 35]. A large part of the recent research focusses on SBO, whereas we included patients with SBO as well as patients with LBO. According to Krouse [38] and other studies [4, 37, 44, 45], bowel resection is a favorable therapy in oncologic patients suffering from MABO in which the malignancy can be removed.

In the trials regarding SBO alone, bowel resection is mostly due to ischemia and thus a marker for an adverse outcome. In our research, the high rate of bowel resection and stoma creation should be regarded in a more differentiated way, considering the rather high rate of MABO in which resection is the recommended therapy if possible.

Furthermore, we regarded the LOS in hospital and the LOS at ICU, as well as the complications classified by the C-D system as secondary outcomes. Similar to van Beekum et al. [30], the LOS of the geriatric patients was significantly longer than that of the non-geriatric patients. We also showed that complications classified by the C-D system were higher in the group of geriatric patients which could explain the longer LOS.

Interestingly, although the time from admission to surgery was no longer in the geriatric patients, the outcome was poorer. This suggests that age, physical status, and comorbidities may be more important regarding the outcome of patients than a single focus on the time from admission to surgery. Regarding time-critical periods, the time from the onset of symptoms to hospital admission, which is more difficult to determine, may be more important for the outcome of patients, because supportive therapy such as fluid resuscitation, decompression via a nasogastric tube, the correction of electrolyte disorders, and antibiotic therapy [9] are immediately initiated when patients are admitted to the hospital, whereas a longer time to admission in which no therapy is carried out could worsen the status of these patients when they arrive at the ED, making emergency surgery more urgent and increasing the risk of mortality. This also means that a worse physical status at admission could shorten the time from admission to surgery because of its urgency.

In the elderly, the time from onset of symptoms to admission may be longer, because geriatric patients are more often in chronic pain or treated with analgesics and notice the onset of new symptoms later or develop fewer symptoms than younger patients. There is some research regarding this time period [46–48] that suggest that the time from the onset of symptoms to admission increases with age [49]. Budzyński et al. [47] showed that the time from the onset of symptoms to admission to hospital was longer in patients with MABO than in patients who suffered from MBO due to adhesions. Furthermore, surgery is more conservatively recommended in the literature for patients suffering from MBO due to malignancies such as peritoneal carcinosis [6, 37, 45]. In addition, the onset of MABO is often described as slow and insidious [4, 8].

For future research, an interesting approach may be to take a closer look at the time from the onset of symptoms to admission to hospital, rather than at the time from admission to surgery.

Given that the population is aging [31] and that the group of geriatric patients showed a significantly higher rate of complications and mortality in our study, we took a closer look at this cohort, searching for particular parameters that affected the outcome of this group (see Table 4).

We discovered that male geriatric patients as well as geriatric patients with a cardiac disease had a significantly longer LOS in the ICU. Like Krause et al. [29], we emphasize that the optimal adjustment of pre-existing cardiac diseases should be carried out before surgery, especially regarding the increasing rate of cardiovascular diseases due to an aging population [32]. This is particularly important for male patients who are more likely to suffer from a cardiac disease [50, 51] and this may reduce their LOS in the ICU.

As we discussed, patients who suffered from a pre-existing malignant disease and from MABO had a higher rate of stoma creation; this confirms that a high proportion of stoma creation in our patients was due to malignancy rather than to delayed surgery. In addition, patients who suffered from MABO had a shorter LOS in the ICU and commonly stayed in a normal ward instead, so early stoma creation may permit the rapid transfer to a normal ward, whereas the LOS in the ICU may depend on pre-existing (especially cardiac) diseases and patients’ general physical conditions instead.

Interestingly, the 30-day mortality rate was significantly higher in the geriatric patients who suffered from LBO. As LBO is caused by a malignancy in 60–70% of cases [8, 14, 36, 37, 45, 52], geriatric patients suffering from LBO may have to undergo more challenging surgery and may be in a worse physical condition due to the underlying disease, leading to higher rates of resection and stoma creation and a higher mortality rate. Therefore, it may be reasonable to carry out the identification of these patients quickly in the ED and to provide special care in view of the high mortality rate.

Beyond that, we are the first to show that even in geriatric patients above the age of 75 years, a watch and wait strategy is justifiable, because the outcome of this group depends on the physical condition and the type of MBO (SBO vs. LBO vs. MABO) rather than on the time from admission to surgery.

The limitations of our study include the retrospective approach and the single institution used for data acquisition. Furthermore, the time between the onset of symptoms and presentation to the ED could not be included in the analysis due to a lack of data in medical records and thus may be an interesting approach for prospective trials.

## Conclusion

This trial emphasizes the finding that geriatric patients, especially those with LBO, are a group in need of special care and are of particular interest for further research in order to aid a reduction in mortality rates. Moreover, focusing on the time between the onset of symptoms and admission rather than on the time between admission and surgery could be an approach for use in future research. Furthermore, our data support the idea that a time period of 3–5 days until surgical treatment in patients suffering from MBO—even in the elderly—is reasonable and does not worsen their outcome. Especially in regard to geriatric patients, clinicians should treat patients in a risk-adapted rather than time-adapted manner, and conditions should be optimized before surgery.

## Supplementary information

Below is the link to the electronic supplementary material.

Supplementary file1 (PDF 154 KB)

## Data Availability

The datasets used and analyzed during the current study are available from the corresponding author on reasonable request.
