# Supplementary material for: Surgical treatment of mechanical bowel obstruction: characteristics and outcomes of geriatric patients compared to a younger cohort
Source: Int J Colorectal Dis. 2022 May 5;37(6):1281–8. doi: 10.1007/s00384-022-04152-4 (PMC9167188; doi:10.1007/s00384-022-04152-4)
Supplement: Supplementary file 1 — Supplementary file1 (PDF 154 KB) [file 384_2022_4152_MOESM1_ESM.pdf]

Additional File 1

Article Title: Surgical treatment of mechanical bowel obstruction: Characteristics and outcomes of geriatric patients compared to a younger cohort.

Journal name: International Journal of Colorectal Disease

Author names: Christian J. J. Paul, Jonas Dohmen, Cornelius J. van Beekum, Maria A. Willis, Lara Braun, Jörg C. Kalff, Arnulf G. Willms and Tim O. Vilz [1]

[1] Department of General-, Visceral-, Thoracic and Vascular Surgery, University Hospital Bonn, Germany.

Email: tim.vilz@ukbonn.de

Supplementary Table 1. Intraoperative findings - SBO

	**40-74 years**	**> =75 years**	**p-value**
	n = 186	n = 71	
Cause of bowel obstruction			
adhesions	107 (57.5%)	50 (70.4%)	ns
all malignancies	39 (21.0%)	9 (12.7%)	ns
colorectal carcinoma	6 (3.2%)	1 (1.4%)	ns
other malignancies	33 (17.7%)	8 (11.3%)	ns
hernia	14 (7.5%)	3 (4.2%)	ns
volvulus	5 (2.7%)	1 (1.4%)	ns
intussusception	3 (1.6%)	2 (2.8%)	ns
gallstone ileus	0 (0.0%)	3 (4.2%)	0,005
inflammation	4 (2.2%)	0 (0.0%)	ns
exposure to radiotherapy	3 (1.6%)	0 (0.0%)	ns
miscellaneous	11 (5.9%)	2 (2.8%)	ns
Need for bowel resection	82 (44.1%)	31 (43.7%)	ns
Need for stoma creation	38 (20.4%)	14 (19.7%)	ns

Supplementary Table 2. Intraoperative findings - LBO

	**40-74 years**	**> =75 years**	**p-value**
	n=28	n=17	
Cause of bowel obstruction			
adhesions	1 (2.6%)	4 (23.5%)	ns
all malignancies	26 (68.4%)	9 (52.9%)	ns
colorectal carcinoma	12 (31.6%)	4 (23.5%)	ns
other malignancies	14 (36.8%)	5 (29.4%)	ns
hernia	1 (2.6%)	1 (5.9%)	ns
volvulus	1 (2.6%)	2 (11.8%)	ns
intussusception	0 (0.0%)	0 (0.0%)	
gallstone ileus	0 (0.0%)	0 (0.0%)	
inflammation	6 (15.8%)	0 (0.0%)	ns
exposure to radiotherapy	1 (2.6%)	0 (0.0%)	
miscellaneous	2 (5.3%)	0 (0.0%)	
Need for bowel resection	25 (65.8%)	13 (76.5%)	ns
Need for stoma creation	33 (86.8%)	10 (58.8%)	ns
